# Molecular evolution and the role of oxidative stress in the expansion and functional diversification of cytosolic glutathione transferases

**DOI:** 10.1186/1471-2148-10-281

**Published:** 2010-09-15

**Authors:** Rute R da Fonseca, Warren E Johnson, Stephen J O'Brien, Vítor Vasconcelos, Agostinho Antunes

**Affiliations:** 1 CIMAR/CIIMAR, Centro Interdisciplinar de Investigação Marinha e Ambiental, Universidade do Porto, Rua dos Bragas, 177, 4050-123 Porto, Portugal; 2 Laboratory of Genomic Diversity, National Cancer Institute, Frederick, MD 21702-1201, USA; 3 Departamento de Biologia, Faculdade de Ciências, Universidade do Porto, Portugal

## Abstract

**Background:**

Cytosolic glutathione transferases (cGST) are a large group of ubiquitous enzymes involved in detoxification and are well known for their undesired side effects during chemotherapy. In this work we have performed thorough phylogenetic analyses to understand the various aspects of the evolution and functional diversification of cGSTs. Furthermore, we assessed plausible correlations between gene duplication and substrate specificity of gene paralogs in humans and selected species, notably in mammalian enzymes and their natural substrates.

**Results:**

We present a molecular phylogeny of cytosolic GSTs that shows that several classes of cGSTs are more ubiquitous and thus have an older ancestry than previously thought. Furthermore, we found that positive selection is implicated in the diversification of cGSTs. The number of duplicate genes per class is generally higher for groups of enzymes that metabolize products of oxidative damage.

**Conclusions:**

1) Protection against oxidative stress seems to be the major driver of positive selection in mammalian cGSTs, explaining the overall expansion pattern of this subfamily;

2) Given the functional redundancy of GSTs that metabolize xenobiotic chemicals, we would expect the loss of gene duplicates, but by contrast we observed a gene expansion of this family, which likely has been favored by: i) the diversification of endogenous substrates; ii) differential tissue expression; and iii) increased specificity for a particular molecule;

3) The increased availability of sequence data from diversified taxa is likely to continue to improve our understanding of the early origin of the different cGST classes.

## Background

Glutathione transferases (GSTs; EC 2.5.1.18) comprise a superfamily of genes encoding ubiquitous enzymes that are very important in the clinical outcome of cancer therapy because they metabolize and inactivate cancer agents (in particular four classes of GST, alpha, mu, pi and theta) [1,2]. Furthermore, the interaction of GSTs with pesticides and pollutants makes them an interesting target for protein engineering in plants [3]. The general role of GSTs in detoxification and the metabolism of xenobiotics has been well documented (reviewed in [3-7]), and makes GSTs a promising subject for evolutionary analyses, especially considering temporal fluctuations in toxic chemicals in the environment.

GSTs are an excellent example of how multiple gene duplication events involving further sub- or neofunctionalization has resulted in groups of enzymes with a myriad of functions. Their occurrence either as homodimers or heterodimers further increases the diversity of the GSTs catalytic activities, which are all centered on chemical reactions that use the tripeptide glutathione (GSH). GSTs are involved in metabolic detoxification of reactive electrophiles, the biosynthesis of leukotrienes, prostaglandins, testosterone and progesterone, and in the degradation of tyrosine [3,5]. Furthermore, some GSTs have also been attributed non-enzymatic regulatory roles [8,9].

The expression of GSTs can be regulated by different stresses (e.g. variation in temperature, oxidative damage, and exposure to toxins) and cGSTs promoters contain antioxidant response elements [3]. This suggests that GSTs could be part of an adaptive response to cellular stress [5,10]. GSH is a scavenger of reactive oxygen species and GSTs are part of the cell machinery responsible for metabolizing by-products of oxidative stress [5]. Indeed, soluble GSTs have been detected in the mitochondria, where they are thought to play an important role in protection against the effects of reactive oxygen species produced by the mitochondrial respiratory chain [11]. However, this protection can also occur when there are cells that are too damaged and should be eliminated, leading to cancer and other diseases [12]. Likewise, a number of GST polymorphisms have been implicated in tumor resistance to chemotherapy [13], and in a number of diseases (Table 1).

**Table 1:** Diseases associated with cGSTs.

GST	Disease	References
**Mu**	Inflammatory diseases*, cancer	[5,42]
**Pi**	Inflammatory diseases*, cancer	[5,43,44]
**Alpha**	Increased susceptibility to bacterial infection	[31]
**Theta**	Inflammatory diseases*, cancer	[1,5]
**Zeta**	Tyrosinemia type I	[45]

*asthma, atherosclerosis, rheumatoid arthritis and systemic sclerosis

GSTs also have noncatalytic roles that include binding (covalently and noncovalently) dangerous chemicals and biomolecules such as bilirubin and hormones [5,14]. Binding to reactive electrophiles is thought to be important for preventing DNA damage whereas for other molecules GSTs act as intracellular carriers [5,14].

Cytosolic GSTs (cGSTs) are by far the most abundant GST subfamily and can be found in all aerobic organisms [3]. They are actively involved in the detoxification of generally nonpolar compounds that contain an electrophilic carbon, nitrogen, or sulfur atom [5]. cGSTs are divided into several classes, the most ubiquitous being alpha (GSTA), mu (GSTM), pi (GSTP), sigma (GSTS), omega (GSTO), zeta (GSTZ) and theta (GSTT) (see Table 2 for the occurrence of these classes in various taxa). These are also the only classes present in mammals. Additional classes are specific to plants (phi and tau), fish (rho), insects (delta and epsilon), and bacteria (beta) [6,15]. The number of isoforms per class varies widely, ranging from one to forty [3].

**Table 2:** Distribution of cGSTs that are present in mammals in major taxa (sequences obtained from GenBank; details in Table 1 in Additional file 1).

	Mu	Pi	Alpha	Sigma	Theta	Zeta	Omega
**Mammals**	X	X	X	X	X	X	X
**Birds**	X		X	X	X	X	X
**Amphibians**	X	X	X	X			
**Fish**	X	X	X		X	X	X
**Mollusks**	X	X		X			
**Insects**				X	X	X	X
**Plants**					X	X	
**Bacteria**			X	X	X	X	X

In this work, we used Bayesian and maximum likelihood-based molecular phylogenetics approaches to ascertain the overall evolutionary pattern in cGSTs. We then focused on mammalian cGSTS and their natural substrates in order to determine if the role of GSTs in detoxification is the major force driving the expansion of this gene family, as it has been suggested [3,5]. More than 20 mammalian cGSTs have been identified to date, many metabolizing the same substrates, especially those substrates of anthropomorphic origin. This catalytic promiscuity probably enhances the formation of duplicates, increasing the probability that duplicated genes become fixed in a population, as adaptation will promote reactions that are already catalyzed by the enzyme [16]. We therefore tested available sequence data for the existence of positive selection using both gene and protein based statistical approaches. Although positive selection has been mostly detected in genes involved in host-pathogen interactions, it has also been shown to influence the active site of enzymes and protein-protein interfaces in membrane receptors [17-19]. The gene-based approach is based on the idea that nonsynonymous substitutions may influence the fitness of an individual or population. Thus, adaptive molecular evolution may cause the nonsynonymous substitution rate (dN) to be higher than the synonymous rate (dS), with the ratio ω (dN/dS) being higher than 1 [20]. Likelihood ratio tests (LRTs) implemented in PAML were used to identify genes under positive selection (ω >1) by comparing two probabilistic models of variable ω ratios among sites, the simpler of which does not allow sites with ω >1 and a more general which does [21]. ConTest was used to measure evolutionary rates in protein sequences accounting for the variation of specific biochemical properties, like volume, polarity and charge [22]. Finally, we intertwined the results from the evolutionary analyzes with information regarding the functions of the different classes of cGSTs, and concluded that the activity of cGSTs on endogenous substrates is sufficient to explain the overall expansion pattern of this subfamily.

## Results & Discussion

### Structure conservation

**Figure 1:**
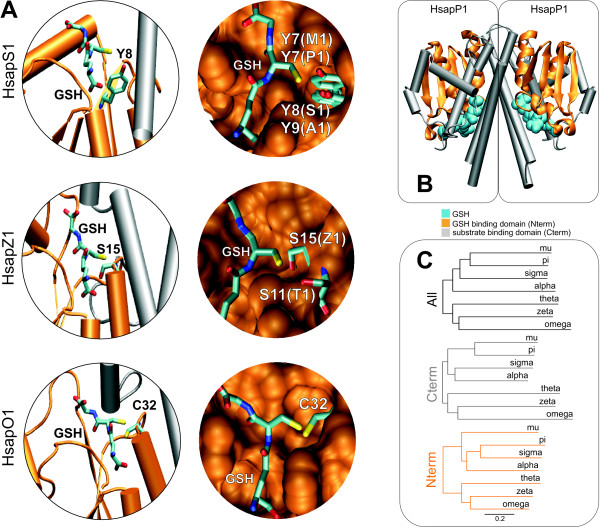
**GSTs structure**. **A**) Active site close-ups showing the different amino acids responsible for activating GSH in different GSTs (left: structures from human GST classes sigma, zeta and omega from top to bottom; right: the amino acids from closely related isoforms (e.g. M1 is GSTM1) are depicted, after superposition of the corresponding structures ); **B) **Structure of a pi GST dimer; **C) **Neighbor-joining trees obtained from distance matrices correspondent to the RMSD in angström between C-alpha carbons of representative structures for each cGST class (Table 4 in Additional file 1).

Within specific GST classes, amino acid sequence identity between paralogues is typically >40%, whereas among classes it can be less than 25% (Table 3 in Additional file 1). cGSTs are characterized by two domains, each containing an active site. The N-terminal domain adopts a thioredoxin-like fold (βαβαββα) that is mostly responsible for binding GSH and the C-terminal domain contains varying numbers of α-helices and encloses the substrate-binding domain (Figure 1). Active cGSTs are either assembled as homodimers or heterodimers formed between elements of the same class (Figure 1B).

**Table 3:** Biological information about the bacterial species present in Figure 3.

Abbreviation	Taxonomic information		Description
**Cwat**	Crocosphaera watsonii	Bacteria; Cyanobacteria; Chroococcales; Crocosphaera	Crocosphaera watsonii is a diazotroph that contributes to the global cycling of nitrogen and carbon through the fixation of atmospheric nitrogen and photosynthesis

**Rpal**	Rhodopseudomonas palustris	Bacteria; Proteobacteria; Alphaproteobacteria; Rhizobiales; Bradyrhizobiaceae; Rhodopseudomonas	Rhodopseudomonas bacteria are purple nonsulfur phototrophic organisms that can be found many types of marine environments and soils. It converts sunlight into energy and converts atmospheric carbon dioxide into biomass. R. palustris can degrade and recycle several aromatic compounds that make up lignin, which makes it useful in removing this type of waste from the environment. In addition, R. palustris converts N2 into NH4 and H2 (used as a biofuel)

**Rhiz**	Rhizobium sp. NGR234	Bacteria; Proteobacteria; Alphaproteobacteria; Rhizobiales; Rhizobiaceae; Rhizobium/Agrobacterium group; Rhizobium.	Rhizobium sp. strain NGR234 is a unique alphaproteobacterium (order Rhizobiales) that forms nitrogen-fixing nodules with more legumes than any other microsymbiont.

**Mnod**	Methylobacterium nodulans	Bacteria; Proteobacteria; Alphaproteobacteria; Rhizobiales; Methylobacteriaceae; Methylobacterium.	Aerobic, facultatively methylotrophic, legume root-nodule-forming and nitrogen-fixing bacteria

**Pstu**	Providencia stuartii	Bacteria; Proteobacteria; Gammaproteobacteria; Enterobacteriales; Enterobacteriaceae	Gram negative bacterium that is commonly found in soil, water, and sewage. It is an opportunistic pathogen seen in patients with severe burns or long-term indwelling urinary catheters. In animals P. stuartii infections can cause neonatal diarrhea due to P stuartii infection in dairy cows.

**Zeta_Hche**	Hahella chejuensis	Bacteria; Proteobacteria; Gammaproteobacteria; Oceanospirillales; Hahellaceae; Hahella	Marine bacteria that produces an algicidal agent (capable of killing phytoplankton, marine eukaryotic microalgae). Suggested as useful for managing algal blooms.

**Vshi**	Vibrio shilonii	Bacteria; Proteobacteria; Gammaproteobacteria; Vibrionales; Vibrionaceae; Vibrio	Vibrio shilonii was isolated from the coral Oculina patagonica in the Mediterranean Sea. This organism cause bleaching (loss of the coral endosymbiotic zooxanthellae). This disease only occurs at elevated seawater temperatures.

**Scel**	Sorangium cellulosum	Bacteria; Proteobacteria; Deltaproteobacteria; Myxococcales; Sorangiineae; Polyangiaceae; Sorangium	Soil-dwelling Gram-negative bacteria of the group myxobacteria. It plays an important role in soil ecology by its ability to degrade cellulosic materials.

**Mxan**	Myxococcus xanthus	Bacteria; Proteobacteria; Deltaproteobacteria; Myxococcales; Cystobacterineae; Myxococcaceae; Myxococcus	Found almost ubiquitously in soil, consists of thin rod shaped, gram-negative cells that exhibit self-organizing behavior as a response to environmental cues. Starving bacteria can self-organize to form dome shaped structures (swarms) of approximately 100,000 cells that, over the course of several days, differentiate into metabolically quiescent and environmentally resistant myxospores.

The N-terminal domain contains residues that are critical for activation of the sulfhydryl group of GSH. Its structure is quite conserved when compared to the substrate binding C-terminal domain (Figure 1C). Conservation in the C-terminal domain is apparent between the elements of the more recently evolved and closely related classes (alpha, mu, pi and sigma), which is in line with their overlapping substrates specificities.

### Phylogeny

**Figure 2:**
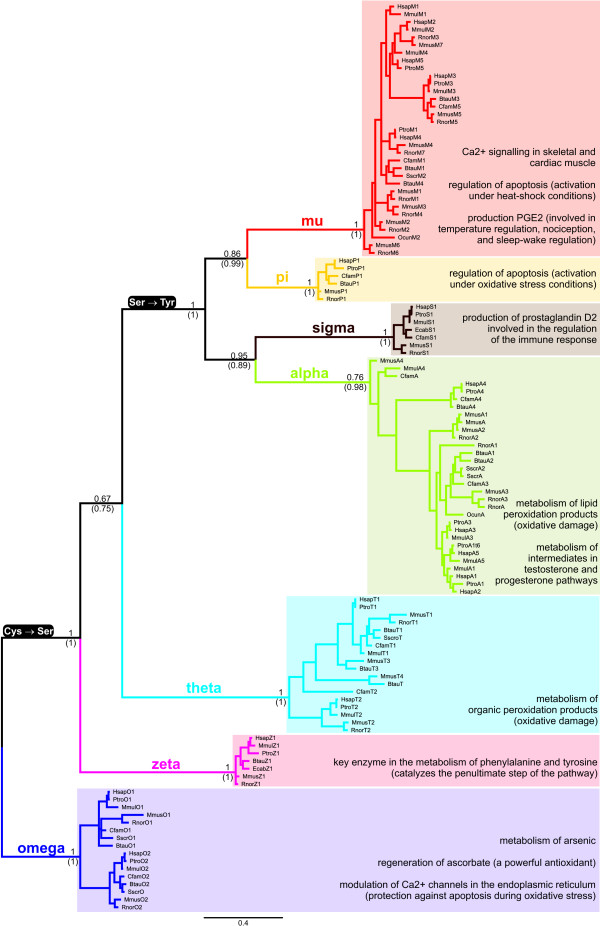
**The nucleotide phylogenetic tree of mammalian cGSTs**. The tree was built in MrBayes, after excluding the third codon position [36]. Posterior probabilities (PP) values are shown (in parenthesis are highlighted the PP values obtained in a tree reconstruction using amino acid sequences). The multiple sequence alignment was first done at the amino acid level using structural information in 3D-Coffee [32]. Changes in the key catalytic amino acid are depicted in black boxes. The enzymatic activities of each class are described on the right. Classes theta, zeta and omega are thought to be the most ancient (see Table 2 for distribution in various taxa).

**Figure 3:**
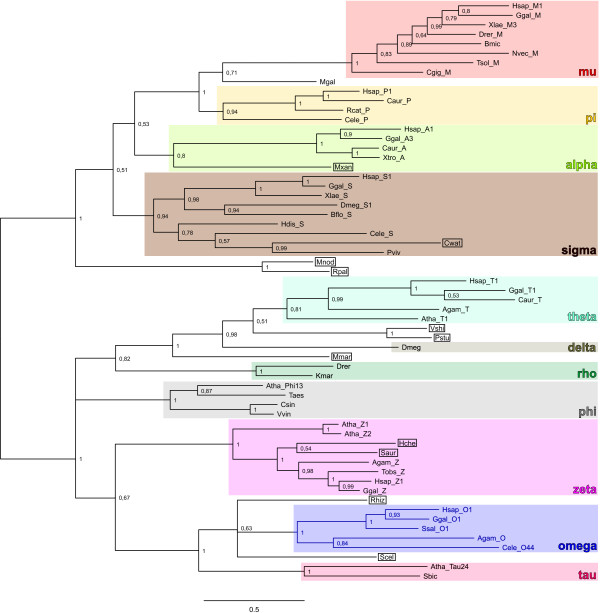
**Phylogenetic tree of cGSTs from various taxonomic groups**. The tree was built in MrBayes [36] and posterior probabilities values are depicted. The multiple sequence alignment was done at the amino-acid level using structural information in PROMALS3D [32]. Bacterial elements are shown in boxes. The first four letter of the sequence name are an abbreviation of the species name and the last characters correspond to the GST isoform, when defined (see Table 1 in Additional file 1).

Given the high sequence divergence within cytosolic GSTs (Table 3 in Additional file 1), building an accurate phylogenetic tree including representatives of all GST gene classes is challenging, and inferences based on these trees should be done cautiously. Since cGSTs protein structure is quite conserved, we have overcome the problems of nucleotide homoplasy by using the structural information when building sequence alignments for cGSTs from multiple classes, which increased confidence in the phylogenetic analyses relative to previous studies (see [6,23] and references therein). The phylogenetic relationship among the cGST classes in mammals is shown in Figure 2. Bayesian and Maximum Likelihood approaches result in the same overall clade topology (Figure 2A in Additional file 1). Orthology and parology are difficult to assess for genes in classes alpha and mu where duplication events were very profuse. The phylogenetic relationships within classes that have a single isoform (pi, sigma and zeta) are incongruent with known phylogenetic relationships, namely for muridae GSTs (Figure 2) [24]. Muridae are also the mammals with the highest number of GST isoforms, another indication that GSTs might have followed an independent evolutionary path in this group. The phylogenetic relationship among the cGST classes in a wide variety of taxa is shown in Figure 3, where we can see a perfect correspondence with the currently accepted phylogeny of species at the animal class level (mammals, amphibians, insects, etc). Theta, zeta and omega cGSTs are generally considered to be the most ancestral, and are found in a wide variety of organisms (Table 2) [3]. However this has been difficult to prove (Figure 3) [3,6]. The omega class are a good choice because they use a cysteine residue to activate GSH (Figure 1A) similarly to glutaredoxins, which are the suggested ancestors of the N-terminal domain of cGSTs [25]. Class theta has been previously appointed as the root of the GST tree mainly because a lot of sequences were initially allocated to this class, and it seemed to be the class represented by the largest diversity of organisms (Table 2). However, this pattern could have been biased in a scenario where some of the classes were lost during evolution because they became nonessential in some species. The fact that class theta is involved in the metabolism of products of oxidative damage could have made it more essential than the other two ancient classes, omega and zeta. The emergence of new genes could also have lead to species-specific redundancy and the elimination of elements of a GST class. Another possibility is that these genes have not been yet detected in certain taxa because of lack of sampling, since more and more GST genes are being detected in newly sequenced genomes. In fact classes alpha and sigma were thought until recently to be only present in metazoans [3,23], but our BLAST searches have successfully retrieved bacterial homologous genes (Figure 3), revealing that the increasing amount of available sequence data is still shaping our knowledge on the evolution of cGSTs. We actually found bacterial homologs for nearly all GST classes (Figure 3), which again shows how GSTs are more ubiquitous than first thought. Even though hypothetical events of horizontal gene-transfer from metazoan to bacteria species are possible, we suggest that these bacterial cGSTs sequences indeed have an ancient origin because: i) there is a deep divergence among bacterial GST sequences (Figure 3), ii) the corresponding organisms inhabit distinct habitats (Table 3), and that iii) these bacteria are phylogenetically divergent (e.g. cyanobacteria vs protobacteria, divergence time over 2 billion years ago [26]; see Figure 1 in Additional file 1).

The cGSTs phylogenetic tree is supported by two fundamental changes in the chemistry of GSTs involving a conserved residue before the third β strand that provides the mainchain hydrogen-bond donors and acceptors for GSH. This residue changes (1) from a cysteine to a serine, and then (2) from a serine to a tyrosine (Figure 1A). This residue activates GSH during catalysis. Classes alpha and mu, which use a tyrosine in the active site, present the higher number of successful duplicates, with up to seven isoforms per species. It is precisely within these classes that we can find extensive signatures of positive selection (Table 4). In the next section we will discuss the functional diversification of mammalian cGSTs considering the relevance of their natural substrates. Also noteworthy is the strong agreement between the phylogenetic tree of the mammalian classes and the tree based on the root mean square deviation (RMSD) between the corresponding structures of the ligand binding domain (Figure 1C).

**Table 4:** Positive selection analysis on mammalian sequences using site models M7 and M8 in PAML [21] (probabilistic models of variable w ratios among sites, the simpler M7 which does not allow sites with w >1 and the more general which does M8).

GST subfamily		p value (M7 vs M8)	
**alpha**	all (31)	4.25E-03	**
	human (5)	6.61E-04	***
	macaque (4)	1.57E-04	***
	mouse (5)	4.48E-04	***
	rat (4)	5.98E-01	ns

**mu**	all (33)	5.02E-06	***
	human (5)	4.32E-04	***
	macaque (4)	4.90E-03	**
	mouse (7)	5.36E-01	ns
	rat (7)	3.58E-03	**

**omega**	all (16)	1.00E+00	ns
	isoform 1 (8)	1.13E-02	*
	isoform 2 (8)	9.21E-01	ns

**pi**	all (6)	9.87e-01	ns

**sigma**	all (7)	3.65E-01	ns

**theta**	all (18)	8.49E-01	ns
	isoform 1 (8)	9.85E-01	ns
	isoform 2 (6)	3.68E-01	ns
	mouse (4)	1.04E-03	**

**zeta**	all (7)	3.26E-01	ns

* 0.01 < p < 0.05; ** 0.001 < p < 0.01; *** p < 0.001; ns: non significant

### Functional diversification and asymmetrical family expansion

The GSTs family has expanded through multiple duplications [3,5]. The fact that GST enzymes accumulate multiple roles, both enzymatic and non-enzymatic, facilitates the process of neofunctionalization by optimization of what was a secondary function [16]. This is obvious for omega cGSTs, where the inference of orthology is pretty straightforward. We suggest that the ancestral enzyme had as a main function the regeneration of ascorbate (the main reaction catalyzed by GSTO2 [27]), since we find that GSTO1 is under positive selection (Table 5). The duplicate has then diverged from this initial function, and optimized what was a secondary reaction in the ancestral enzyme, the metabolism of arsenic. We expect this pattern to be general in GSTs: duplicates sometimes retain the previous main function but are optimized for a new one (e.g. both GSTOs are involved in the metabolism of arsenic and ascorbate, but GSTO1 is better in metabolizing arsenic and GSTO2 has an activity 70-100 times greater than GSTO1 towards ascorbate [27]).

**Table 5:** Sites under positive selection detected by the BEB approach in PAML (bold, PP >0.9) and by ConTest (underlined, p < 0.05).

Alpha	Mu	Omega	Theta	Location	Amino acids	Experimentally confirmed functional relevance
**12**				interdomain interface	G, A, V, I, T	

**36**				C-terminal domain interface	G, N, Q, K, E, H	

	**104**			substrate binding pocket	A, V, L, I, T, F	

**103**				substrate binding pocket	G, L, M, T, N, E	

		**128**		substrate binding pocket	G, A, L, I, T, R	

**111**				substrate binding pocket	V, L, I, T, F, Y , H	Residue 110 [46]

**112**					I, C, M, T, D, K	[46]

	**126**			interdomain interface	G, Q, R, K, E	

	**129**			substrate binding pocket	Q, K, E	

	**130**				G, A, T, S, Q, E	

	**166**			substrate binding pocket	L, I, M, Q, R	

			**186**	substrate binding pocket	V, L, E, K, R,	

	**192**			N-terminal domain interface	K, E	

			**204**	substrate binding pocket	A, S, K, R, E	

**208**				substrate binding pocket	A, V, L, M, T, D, E, P	

	**210**			interdomain interface	G, A, L, M, S, T, N	[30,47]

**212**				interdomain interface	A, S, N, Q, K, Y	Residue 210 [46]

	**214**			substrate binding pocket	V, I, M, T, Q, K, F, H	

			**234**	C-terminal	S, R	[48]

	**218**				I, M, T, N, K, R, E, D	

			**237**	C-terminal	A, L, C, T, D	

Numbering is according to the human sequence of variant 1 for each subfamily.

The expansion of the cGSTs was particularly profuse, but as we can see in mammals, not all the resulting classes of enzymes have duplicated extensively. For example, sigma and zeta cGSTs are involved in pathways that require a more precise regulation, and for which the existence of a duplicate could have been harmful. GSTZ is a key enzyme in the metabolic degradation of phenylalanine and tyrosine and its product can cause the fatal hereditary disease tyrosinemia type I [28]. GSTS produces prostaglandin D_2 _[29] (prostaglandins are lipid mediators that are involved in the regulation of the respiratory, cardiovascular, central nervous system (CNS), genitourinary, endocrine, and immune systems). In contrast, alpha, mu and theta cGSTs are all involved in the cellular reaction under stress conditions, and have multiple duplicates (up to seven, five and four duplicates for alpha, mu and theta classes, respectively, compared to only one isoform in e.g. sigma and zeta classes) that probably contributed to an increase in fitness by the elimination of a broader range of reactive and harmful chemicals, especially through variation in the substrate binding pocket. Changes of a hypervariable site in this region have been shown to have a central role in defining enzyme specificity [30]. Our hypothesis is thus supported by evidence of several positively selected sites in the substrate binding pocket (Table 5).

The catalytic promiscuity of GSTs has certainly facilitated the process of neofunctionalization and duplicate gene retention for the most populated cGST classes. We further suggest that subsequent loss of duplicates arising from functional redundancy was probably avoided by variation in tissue expression (e.g. human GSTA3 is expressed solely in steroidogenic tissues and GSTM3 is selectively expressed in testis and brain [31]) and increased specificity for a particular molecule (such as GSTO1 that has an improved efficiency in metabolizing arsenic, see above).

## Conclusions

Glutathione transferases enzymes represent a superfamily with many functional roles throughout evolution. This work has revealed that cGSTs are a lot more ubiquitous and old than previously thought, with many cGSTs classes having a bacterial isoform. The need for protection against the products of oxidative damage has no doubt driven the expansion of the family via positive selection on GST duplicates, but on the way GSTs have acquired many other roles such as the metabolism of sex hormones and the regulation of apoptosis, which likely were vital for the retention of duplicates. The metabolism of dangerous xenobiotics is also a major role of GSTs and has been suggested to be driving the expansion of the family. In fact, times when changes in the levels and the toxicity of environmentally available chemicals have occurred, the catalytic promiscuity and an elevated number of sGST isoforms would have constituted a fitness advantage. However, the functional redundancy towards many of these chemicals would eventually lead to a loss in the number of cGST isoforms. We suggest that the expansion of the cGST family was strongly favored by the fact that the different isoforms within a class have acquired new functions towards endogenous substrates. We hypothesize that these roles dictate the number of duplicates per class, being more abundant for classes involved in the metabolism of product of oxidative stress (alpha, mu and theta), but reduced to one when related with tightly regulated biological processes (such is the case for class zeta involved in the metabolism of the aromatic amino acids tyrosine and phenylalanine). The need for duplicates with sometimes overlapping substrate specificities is further supported by differences in tissue specificity and the increase of specificity for a particular reaction that is weakly catalyzed by other isoforms.

Presently it is still difficult to ascertain the most ancient class of cGSTs, with theta and omega being the best candidates. Nevertheless, the information arising from whole-genome sequencing of an increasing number of non-mammalian species will certainly provide further insight into the origins and evolution of this ancient gene family.

## Materials and methods

The cGST sequences used in this work were obtained from GenBank and are listed in Table 1 in Additional file 1.

Because of the high sequence divergence of cGSTs, amino acid multiple sequence alignments were done using protein structure information in 3D-Coffee [32] and PROMALSD [33](see Additional Files 2 and 3 for the alignments used to build the trees presented on Figures 2 and 3, respectively). The codons in the nucleotide alignment were aligned accordingly. We have chosen a limited number of representative sequences for all clades (the insect epsilon class is omitted as it forms a clade with the delta class). DAMBE and MEGA4 were used for sequence editing and formatting [34,35].

We have built the various phylogenetic trees based on the nucleotide and amino acid alignments using MrBayes [36] and PhyML [37] after determining the optimal model of sequence substitution with Modeltest 3.04 (TVM+I+G) [38] and Prottest (JTT+I+G) [39]. One cold and four incrementally heated chains were run for 2,000,000 generations with chains I = 2, 3, 4, and 5 incrementally heated with heat being 1/(1+[i-1]T) and T = 0.2. Following a burn-in of 500,000, trees were sampled every 100 generations (well after the chain reached stationarity) and 15,000 trees were used for inferring Bayesian posterior probability. The nucleotide tree was obtained after removal of the third codon position. The trees obtained with PhyML are presented in Figure 2 in Additional file 1. All trees were drawn in FigTree http://tree.bio.ed.ac.uk/software/figtree/.

Likelihood ratio tests (LRTs) implemented in PAML [21] were used to identify genes under positive selection. These tests are used to identify adaptive molecular evolution which occurs when the nonsynonymous substitution rate (dN) is higher than the synonymous rate (dS), with the ratio ω (dN/dS) being higher than 1 [20]. The LRTs used compare two probabilistic models of variable ω ratios among sites, the simpler of which does not allow sites with ω >1 and a more general which does [21] (model M7 *vs *M8). Amino acid sites under positive selection were detected with Bayesian empirical bayes (BEB) inference under the M8 model in PAML [21], and with CONTEST, that assesses protein changes in biochemical constraints to calculate evolutionary rates [22]. CONTEST accounts for the variation of specific biochemical properties, like volume, polarity and charge and presents a new statistical method based on the comparison of two-rate measures where a site is considered constrained for a given property if it shows high conservation relatively to its total evolutionary rate (some positions may be constrained while having a high substitution rate, provided these substitutions do not affect the biochemical property under constraint). CONTEST can also be used to infer positively selected positions, as it looks for sites that have experienced more nonconservative substitutions than expected by chance under the neutral hypothesis. Sequence alignments were built to perform the positive selection test. For classes where orthology/parology was difficult to assign, tests were done using sets of genes existing in single species (human, macaque, mouse and rat; Table 4 and Table 5).

Multiprot [40] was used to calculate the root mean square deviation (RMSD) between C-alpha atoms of cGST structures (Table 2 in Additional file 1). Neighbor-joining trees were obtained from the corresponding distance matrices in PAUP [41].

## List of abbreviations used

GST: glutathione transferase; GSH: glutathione; GSTA: GST alpha; GSTM: GST mu; GSTP: GST pi; GSTS: GST sigma; GSTO: GST omega; GSTZ: GST zeta; GSTT: GST theta; AGAM: *Anopheles gambiae*; ATHA: *Arabidopsis thaliana*; BFLO: *Branchiostoma floridae*; BMIC: *Boophilus microplus*; BTAU: *Bos Taurus*; CAUR: *Carassius auratus*; CELE: *Caenorhabditis elegans*; CFAM: *Canis familiaris*; CGIG: *Crassostrea gigas*; CSIN: *Citrus sinensis*; CWAT: *Crocosphaera watsonii*; DMEL: *Drosophila melanogaster*; DRER: *Danio rerio*; ECAB: *Equus caballus*; GGAL: *Gallus gallus*; Hche: *Hahella chejuensis*; HDIS: *Haliotis discus discus*; HLON: *Haemaphysalis longicornis*; HSAP: *Homo sapiens*; KMAR: *Kryptolebias marmoratus*; MGAL: *Mytilus galloprovincialis*; MMAR: *Maricaulis maris*; MMUL: *Macaca mulatta*; MMUS: *Mus musculus*; MNOD: *Methylobacterium nodulans*; MXAN: *Myxococcus Xanthus*; NVEC: *Nematostella vectensis*; OCUN: *Oryctolagus cuniculus*; OSLO: *Ommastrephes sloani*; PSTU: *Providencia stuartii*; PTRO: *Pan troglodytes*; PVIV: *Plasmodium vivax*; RCAT: *Rana catesbeiana*; RHIZ: *Rhizobium sp. NGR234*; RNOR: *Rattus norvegicus*; RPAL: *Rhodopseudomonas palustris*; SAUR: *Stigmatella aurantiaca*; SBIC: *Sorghum bicolor*; SCEL: *Sorangium cellulosum*; SSAL: *Salmo salar*; SSCR: *Sus scrofa*; TAES: *Triticum aestivum*; TOBS: *Takifugu obscures*; TSOL: *Taenia solium*; VSHI: *Vibrio shilonii*; VVIN: *Vitis vinifera*; XLAE: *Xenopus laevis*; XTRO: *Xenopus tropicalis*.

## Authors' contributions

RF performed all phylogenetic, evolutionary and structure-function analyses and drafted the manuscript.

WEJ participated in the drafting and coordination of the study.

SJOB participated in the drafting and coordination of the study.

VV participated in the drafting and coordination of the study.

AA participated in the design, genetic analyses, drafting and coordination of the study.

All authors read and approved the final manuscript.

## Supplementary Material

Additional file 1 **Additional figures and tables**. Additional figures and tables. Click here for file

Additional file 2 **Alignment Figure 2**. Alignment used to build the tree presented on Figure 2 Click here for file

Additional file 3 **Alignment Figure 3**. Alignment used to build the tree presented on Figure 3 Click here for file
