# Supplementary material for: Molecular evolution and the role of oxidative stress in the expansion and functional diversification of cytosolic glutathione transferases
Source: BMC Evol Biol. 2010 Sep 15;10:281. doi: 10.1186/1471-2148-10-281 (PMC2955027; doi:10.1186/1471-2148-10-281)
Supplement: Additional file 1 — Additional figures and tables. Additional figures and tables. [file 1471-2148-10-281-S1.DOC]

**Table 1.** GST sequences used in this work.

| **Type** | **class** | **isoform** | **abbrev** | **species name** | **GI** |
| --- | --- | --- | --- | --- | --- |
| alveolata | sigma |  | Pviv | Plasmodium vivax | 76160839 |
| amphibian | pi |  | Rcat | Rana catesbeiana | 226372468 |
| amphibian | mu | 2 | Xlae | Xenopus laevis | 147905958 |
| amphibian | sigma |  | Xlae | Xenopus laevis | 147899575 |
| amphibian | alpha |  | Xtro | Xenopus tropicalis | 163914809 |
| arthropoda | mu |  | Bmic | Boophilus microplus | 4322274 |
| arthropoda | pi |  | Hlon | Haemaphysalis longicornis | 34539115 |
| bacteria |  |  | Cwat | Crocosphaera watsonii | 67922776 |
| bacteria |  |  | Hche | Hahella chejuensis | 83643830 |
| bacteria |  |  | Mmar | Maricaulis maris | 114570725 |
| bacteria |  |  | Mnod | Methylobacterium nodulans | 220926485 |
| bacteria |  |  | Mxan | Myxococcus xanthus | 108761472 |
| bacteria |  | 44 | Pstu | Providencia stuartii | 183598565 |
| bacteria |  |  | Rhiz | Rhizobium sp. NGR234 | 227824143 |
| bacteria |  |  | Rpal | Rhodopseudomonas palustris | 115523581 |
| bacteria |  |  | Saur | Stigmatella aurantiaca | 115377235 |
| bacteria |  |  | Scel | Sorangium cellulosum | 162448872 |
| bacteria |  |  | Vshi | Vibrio shilonii | 149187624 |
| bird | alpha | 3 | Ggal | Gallus gallus | 45382235 |
| bird | sigma |  | Ggal | Gallus gallus | 45384344 |
| bird | zeta |  | Ggal | Gallus gallus | 118091881 |
| bird | theta | 1 | Ggal | Gallus gallus | 45382479 |
| bird | mu |  | Ggal | Gallus gallus | 2506495 |
| bird | omega | 1 | Ggal | Gallus gallus | 50749765 |
| Cephalochordata (amphioxus) | sigma |  | Bflo | Branchiostoma floridae | 260831252 |
| cnidaria | mu |  | Nvec | Nematostella vectensis | 156401515 |
| fish | pi |  | Caur | Carassius auratus | 159884969 |
| fish | theta |  | Caur | Carassius auratus | 159191983 |
| fish | alpha |  | Caur | Carassius auratus | 158347524 |
| fish | mu |  | Drer | Danio rerio | 182890420 |
| fish | rho |  | Drer | Danio rerio | 113682261 |
| fish | theta |  | Kmar | Kryptolebias marmoratus | 102230838 |
| fish | omega | 1 | Ssal | Salmo salar | 213511516 |
| fish | zeta |  | Tobs | Takifugu obscurus | 157152701 |
| insect | omega |  | Agam | Anopheles gambiae | 58387722 |
| insect | zeta |  | Agam | Anopheles gambiae | 58382539 |
| insect | theta |  | Agam | Anopheles gambiae | 58389215 |
| insect | sigma |  | Dmel | Drosophila melanogaster | 24654347 |
| insect | delta |  | Dmel | Drosophila melanogaster | 17737923 |
| mammal | zeta | 1 | Btau | Bos taurus | 115496801 |
| mammal | alpha | 4 | Btau | Bos taurus | 62751788 |
| mammal | alpha | 1 | Btau | Bos taurus | 118151371 |
| mammal | alpha | 2 | Btau | Bos taurus | 92096974 |
| mammal | theta | 3 | Btau | Bos taurus | 115496313 |
| mammal | theta | 1 | Btau | Bos taurus | 114052950 |
| mammal | mu | 1 | Btau | Bos taurus | 73853765 |
| mammal | mu | 3 | Btau | Bos taurus | 114053086 |
| mammal | mu | 4 | Btau | Bos taurus | 122692370 |
| mammal | theta |  | Btau | Bos taurus | 194674442 |
| mammal | omega | 2 | Btau | Bos taurus | 194678797 |
| mammal | omega | 1 | Btau | Bos taurus | 164420718 |
| mammal | pi | 1 | Btau | Bos taurus | 29135328 |
| mammal | mu | 5 | Cfam | Canis familiaris | 73959314 |
| mammal | mu | 1 | Cfam | Canis familiaris | 73959316 |
| mammal | sigma | 1 | Cfam | Canis familiaris | 74002251 |
| mammal | theta | 2 | Cfam | Canis familiaris | 73995690 |
| mammal | theta | 1 | Cfam | Canis familiaris | 73995859 |
| mammal | alpha | 3 | Cfam | Canis familiaris | 73973353 |
| mammal | alpha | 4 | Cfam | Canis familiaris | 74001085 |
| mammal | pi | 1 | Cfam | Canis familiaris | 74007130 |
| mammal | omega | 2 | Cfam | Canis familiaris | 73998805 |
| mammal | omega | 1 | Cfam | Canis familiaris | 73998493 |
| mammal | alpha |  | Cfam | Canis familiaris | 73973356 |
| mammal | sigma | 1 | Ecab | Equus caballus | 149701564 |
| mammal | zeta | 1 | Ecab | Equus caballus | 149737404 |
| mammal | mu | 1 | Hsap | Homo sapiens | 215276995 |
| mammal | mu | 3 | Hsap | Homo sapiens | 215277001 |
| mammal | mu | 2 | Hsap | Homo sapiens | 215276998 |
| mammal | mu | 5 | Hsap | Homo sapiens | 215277008 |
| mammal | mu | 4 | Hsap | Homo sapiens | 183662 |
| mammal | omega | 2 | Hsap | Homo sapiens | 168743729 |
| mammal | omega | 1 | Hsap | Homo sapiens | 31873363 |
| mammal | pi | 1 | Hsap | Homo sapiens | 31945 |
| mammal | zeta | 1 | Hsap | Homo sapiens | 194394144 |
| mammal | alpha | 5 | Hsap | Homo sapiens | 151555562 |
| mammal | alpha | 4 | Hsap | Homo sapiens | 215276994 |
| mammal | alpha | 1 | Hsap | Homo sapiens | 215276985 |
| mammal | alpha | 3 | Hsap | Homo sapiens | 215276991 |
| mammal | alpha | 2 | Hsap | Homo sapiens | 123999323 |
| mammal | sigma | 1 | Hsap | Homo sapiens | 158261172 |
| mammal | theta | 2 | Hsap | Homo sapiens | 50482556 |
| mammal | theta | 1 | Hsap | Homo sapiens | 167466163 |
| mammal | sigma | 1 | Mmul | Macaca mulatta | 109075012 |
| mammal | alpha | 4 | Mmul | Macaca mulatta | 109071558 |
| mammal | alpha | 5 | Mmul | Macaca mulatta | 109071516 |
| mammal | alpha | 1 | Mmul | Macaca mulatta | 109071510 |
| mammal | alpha | 3 | Mmul | Macaca mulatta | 109071518 |
| mammal | omega | 2 | Mmul | Macaca mulatta | 109090493 |
| mammal | omega | 1 | Mmul | Macaca mulatta | 109131385 |
| mammal | mu | 1 | Mmul | Macaca mulatta | 109013312 |
| mammal | mu | 2 | Mmul | Macaca mulatta | 109013300 |
| mammal | mu | 3 | Mmul | Macaca mulatta | 109013330 |
| mammal | mu | 4 | Mmul | Macaca mulatta | 109013273 |
| mammal | zeta | 1 | Mmul | Macaca mulatta | 109084409 |
| mammal | theta | 2 | Mmul | Macaca mulatta | 109043009 |
| mammal | theta | 1 | Mmul | Macaca mulatta | 109094859 |
| mammal | alpha | 3 | Mmus | Mus musculus | 187957677 |
| mammal | alpha | 2 | Mmus | Mus musculus | 118130920 |
| mammal | alpha | 1 | Mmus | Mus musculus | 38173960 |
| mammal | alpha | 4 | Mmus | Mus musculus | 160298216 |
| mammal | alpha |  | Mmus | Mus musculus | 20988788 |
| mammal | theta | 1 | Mmus | Mus musculus | 12832244 |
| mammal | theta | 2 | Mmus | Mus musculus | 158081795 |
| mammal | theta | 3 | Mmus | Mus musculus | 133892397 |
| mammal | theta | 4 | Mmus | Mus musculus | 227330607 |
| mammal | omega | 1 | Mmus | Mus musculus | 133892764 |
| mammal | omega | 2 | Mmus | Mus musculus | 12858931 |
| mammal | zeta | 1 | Mmus | Mus musculus | 133892597 |
| mammal | pi | 1 | Mmus | Mus musculus | 38173954 |
| mammal | mu | 7 | Mmus | Mus musculus | 113679873 |
| mammal | mu | 6 | Mmus | Mus musculus | 113680505 |
| mammal | mu | 5 | Mmus | Mus musculus | 133892313 |
| mammal | mu | 4 | Mmus | Mus musculus | 238018079 |
| mammal | mu | 3 | Mmus | Mus musculus | 33468898 |
| mammal | mu | 2 | Mmus | Mus musculus | 141802539 |
| mammal | mu | 1 | Mmus | Mus musculus | 239937552 |
| mammal | sigma | 1 | Mmus | Mus musculus | 26333236 |
| mammal | alpha |  | Ocun | oryctolagus cuniculus | 349537 |
| mammal | mu | 2 | Ocun | oryctolagus cuniculus | 126722905 |
| mammal | mu | 1 | Ptro | Pan troglodytes | 114558269 |
| mammal | mu | 3 | Ptro | Pan troglodytes | 114559052 |
| mammal | mu | 5 | Ptro | Pan troglodytes | 114559055 |
| mammal | theta | 2 | Ptro | Pan troglodytes | 114687358 |
| mammal | theta | 1 | Ptro | Pan troglodytes | 114685411 |
| mammal | alpha | 1t6 | Ptro | Pan troglodytes | 114607874 |
| mammal | zeta | 1 | Ptro | Pan troglodytes | 114654141 |
| mammal | sigma | 1 | Ptro | Pan troglodytes | 114595207 |
| mammal | alpha | 4 | Ptro | Pan troglodytes | 114607890 |
| mammal | alpha | 1 | Ptro | Pan troglodytes | 114607852 |
| mammal | alpha | 3 | Ptro | Pan troglodytes | 114607882 |
| mammal | pi | 1 | Ptro | Pan troglodytes | 114638782 |
| mammal | omega | 2 | Ptro | Pan troglodytes | 114632708 |
| mammal | omega | 1 | Ptro | Pan troglodytes | 114632702 |
| mammal | zeta | 1 | Rnor | Rattus norvegicus | 208969734 |
| mammal | theta | 2 | Rnor | Rattus norvegicus | 38197387 |
| mammal | mu | 7 | Rnor | Rattus norvegicus | 66730312 |
| mammal | theta | 1 | Rnor | Rattus norvegicus | 208969730 |
| mammal | mu | 6 | Rnor | Rattus norvegicus | 165970857 |
| mammal | mu | 4 | Rnor | Rattus norvegicus | 10120485 |
| mammal | mu | 5 | Rnor | Rattus norvegicus | 25282394 |
| mammal | mu | 2 | Rnor | Rattus norvegicus | 208969718 |
| mammal | mu | 3 | Rnor | Rattus norvegicus | 18043912 |
| mammal | mu | 1 | Rnor | Rattus norvegicus | 38648906 |
| mammal | alpha |  | Rnor | Rattus norvegicus | 1835950 |
| mammal | pi | 1 | Rnor | Rattus norvegicus | 208969728 |
| mammal | omega | 1 | Rnor | Rattus norvegicus | 56090549 |
| mammal | omega | 2 | Rnor | Rattus norvegicus | 208969726 |
| mammal | alpha | 2 | Rnor | Rattus norvegicus | 208969708 |
| mammal | alpha | 3 | Rnor | Rattus norvegicus | 208969710 |
| mammal | alpha | 1 | Rnor | Rattus norvegicus | 66730520 |
| mammal | sigma | 1 | Rnor | Rattus norvegicus | 56799410 |
| mammal | alpha | 2 | Sscr | Sus scrofa | 47523831 |
| mammal | theta |  | Sscr | Sus scrofa | 115547351 |
| mammal | omega | 1 | Sscr | Sus scrofa | 52351223 |
| mammal | mu | 2 | Sscr | Sus scrofa | 118403787 |
| mammal | omega |  | Sscr | Sus scrofa | 194041987 |
| mammal | alpha |  | Sscr | Sus scrofa | 47523157 |
| mollusca | mu |  | Cgig | Crassostrea gigas | 47076115 |
| mollusca | sigma | 2 | Hdis | Haliotis discus discus | 126697292 |
| mollusca |  |  | Mgal | Mytilus galloprovincialis | 22094809 |
| mollusca | sigma |  | Oslo | Ommastrephes sloani | 1170111 |
| mollusca | sigma |  | Oslo | Ommastrephes sloani | 1170111 |
| nematoda | pi |  | Cele | Caenorhabditis elegans | 6749 |
| nematoda |  | 5 | Cele | Caenorhabditis elegans | 17534681 |
| nematode | omega | 44 | Cele | Caenorhabditis elegans | 193207701 |
| plant | phi | 13 | Atha | Arabidopsis thaliana | 15228853 |
| plant | zeta | 2 | Atha | Arabidopsis thaliana | 15226949 |
| plant | zeta | 1 | Atha | Arabidopsis thaliana | 42570653 |
| plant | tau | 24 | Atha | Arabidopsis thaliana | 15220040 |
| plant | theta | 1 | Atha | Arabidopsis thaliana | 15237583 |
| plant | phi |  | Csin | Citrus sinensis | 76365793 |
| plant | tau |  | Sbic | Sorghum bicolor | 242094102 |
| plant | phi | F6 | Taes | Triticum aestivum | 23504747 |
| plant | phi |  | Vvin | Vitis vinifera | 62361403 |
| platyhelminthe | mu |  | Tsol | Taenia solium | 21591409 |

**Table 2.** PDB structures used to build the distance matrices used in Figure 2.

| **cGST** | **PDB id** |
| --- | --- |
| Alpha | 1PKW:A |
| Mu | 2F3M:E |
| Pi | 9GSS:A |
| Sigma | 2VCQ:A |
| Theta | 2C3Q:A |
| Omega | 1EEM:A |

**Table 3.** Percent sequence identities between the different cGST classes determined in MatGAT, http://bitincka.com/ledion/matgat/ (alpha: Hsap_A1; pi: Hsap_P1; sigma: Dmeg_S1; phi: Atha_Phi13; zeta: Agam_Z; theta: Atha_T1; delta: Dmeg_Delta; tau: Atha_Tau24; omega: Agam_O; rho: Drer_Rho; see Table 1 in Supplementary Material for abbreviations)

|  | pi | sigma | phi | zeta | theta | delta | tau | omega | rho |
| --- | --- | --- | --- | --- | --- | --- | --- | --- | --- |
| alpha | 29 | 23 | 25 | 22 | 23 | 22 | 21 | 23 | 20 |
| pi |  | 25 | 21 | 23 | 22 | 17 | 15 | 17 | 20 |
| sigma |  |  | 19 | 15 | 17 | 17 | 18 | 16 | 20 |
| phi |  |  |  | 28 | 29 | 24 | 23 | 25 | 25 |
| zeta |  |  |  |  | 23 | 21 | 25 | 26 | 23 |
| theta |  |  |  |  |  | 30 | 17 | 25 | 24 |
| delta |  |  |  |  |  |  | 23 | 22 | 24 |
| tau |  |  |  |  |  |  |  | 24 | 26 |
| omega |  |  |  |  |  |  |  |  | 21 |


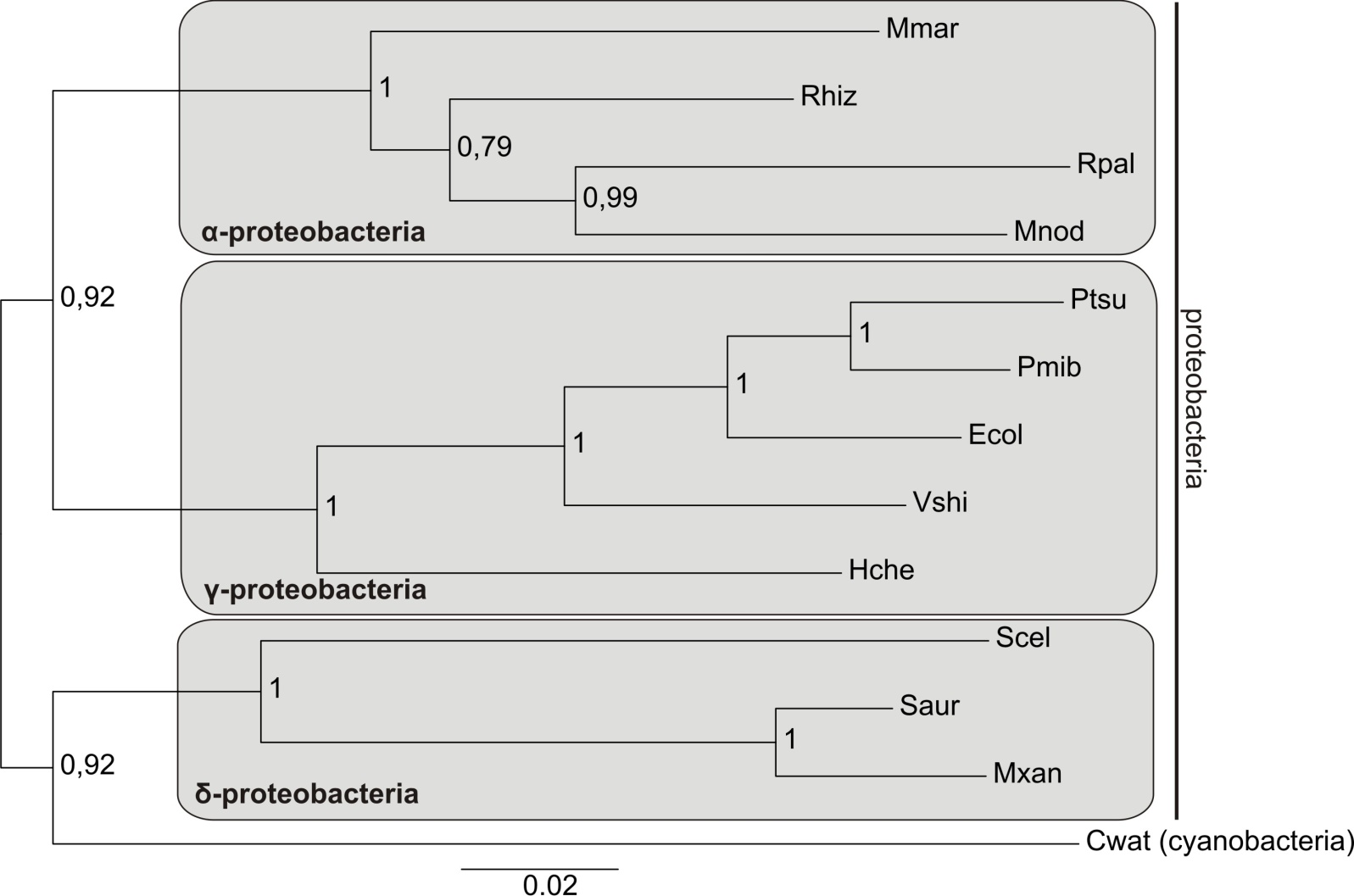


**Figure 1.** Phylogenetic relationships between the bacteria in Table 1 established using the 16S gene (GIs: Cwat:53987838; Mmar; 4138231; Rpal: 256576992; Rhi: 30144675; Mnod: 12239376; Ptsu: 6478167; Hche: 13873309; Vshi: 260223563; Ecol: 282896634; Pmib; 251825134; Scel: 160221099; Saur: 110564227; Mxan: 166033767). NJ tree built in MEGA (bootstrap values for 10000 replicates are shown).


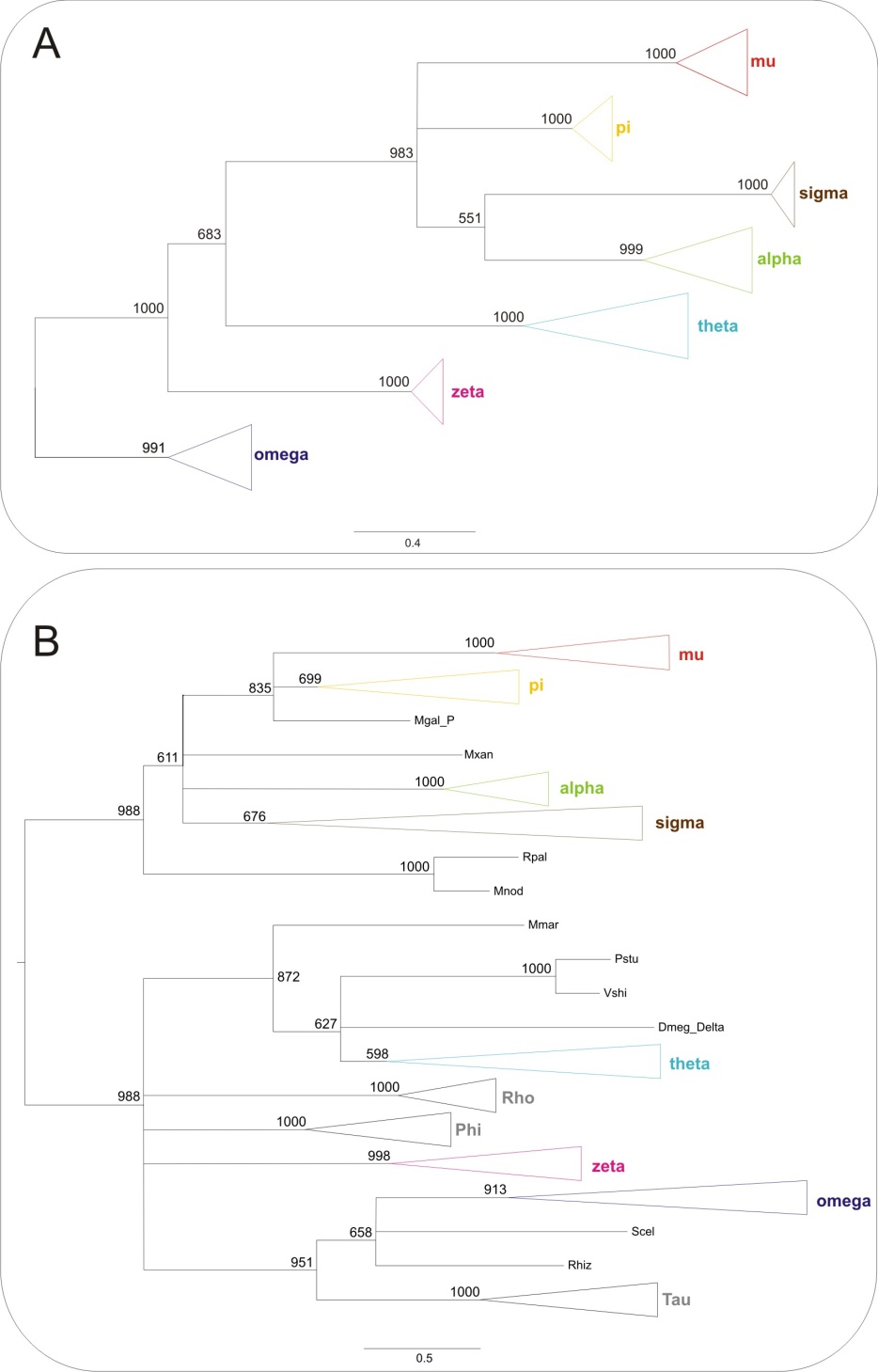


**Figure 2.** Phylogenetic trees of cGSTs built in PhyML. A) mammalian nucleotide tree obtained after excluding the third codon position; B) amino acid phylogenetic tree of cGSTs from various taxonomic groups, representing several known cGST classes.
